# Evolution of Power Losses in Bending Rolled Fully Finished NO Electrical Steel Treated under Unconventional Annealing Conditions

**DOI:** 10.3390/ma12132200

**Published:** 2019-07-08

**Authors:** Ivan Petryshynets, František Kováč, Ján Füzer, Ladislav Falat, Viktor Puchý, Peter Kollár

**Affiliations:** 1 Institute of Materials Research, Slovak Academy of Sciences, Watsonova 47, 04001 Košice, Slovakia; 2 Institute of Physics, Faculty of Science, Pavol Jozef Safarik University, Park Angelinum 9, 041 54 Košice, Slovakia

**Keywords:** electrical steel, crystallographic texture, magnetic losses, bending deformation

## Abstract

Currently, the non-oriented (NO) iron-silicon steels are extensively used as the core materials in various electrical devises due to excellent combination of their mechanical and soft magnetic properties. The present study introduces a fairly innovative technological approach applicable for fully finished NO electrical steel before punching the laminations. It is based on specific mechanical processing by bending and rolling in combination with subsequent annealing under dynamic heating conditions. It has been revealed that the proposed unconventional treatment clearly led to effective improvement of the steel magnetic properties thanks to its beneficial effects involving additional grain growth with appropriate crystallographic orientation and residual stress relief. The philosophy of the proposed processing was based on employing the phenomena of selective grain growth by strain-induced grain boundary migration and a steep temperature gradient through the cross-section of heat treated specimens at dynamic heating conditions. The stored deformation energy necessary for the grain growth was provided by plastic deformation induced within the studied specimens during the bending and rolling process. The magnetic measurements clearly show that the specimens treated according to our approach exhibited more than 17% decrease in watt losses in comparison with the specimens treated by conventional heat treatment leading only to stress relief without additional grain growth.

## 1. Introduction

Increasing demands of modern society on the efficient exploitation of various energy sources as well as protection of global environment invoke permanent pressure on continuing development of new technologies and materials. In recent years, humankind has actively begun to use various sources of renewable and clean energy. At present, the only way that guarantees the easy and effective use of these energy sources is related to their transformation to electric power and its subsequent distribution to final consumers. The technologies that are pursued for increasing the efficiency of distribution, transformation, and end-use of electricity depend on the research and development of materials that are used as core segments in electrical motors, transformers, and generators. These materials are represented by electrical silicon steels, which are generally classified into two categories, namely the grain-oriented (GO) and non-oriented (NO) electrical steels [1,2,3]. 

The NO electrical steels belong to the group of soft magnetic materials, which are easily magnetized and demagnetized [4]. These steels are mainly used in conditions of alternating current (AC) electromagnetic field at a frequency of 50 Hz (or 60 Hz) and represent the base materials for industrial production of the cores for asynchronous motors, large electric rotating machines, small generators, and other electrical motors, which transform electrical energy into mechanical or vice versa [5]. Generally required electromagnetic properties of electrical steels are high magnetic induction, high magnetic permeability, and low core loss [6]. The core loss of NO electrical steel can be separated into its partial contributions, such as hysteresis loss, classical eddy-current loss, and excess loss. Magnetic properties of electrical steel, especially the hysteresis loss and excess loss, are known to be affected by the structure and behavior of magnetic domains under external fields [7,8,9] and related to the microstructure and texture of the steel. The microstructural parameters that have a strong influence on the magnetic properties are grain size, inclusions, internal stresses, and surface defects. These microstructural parameters determine the domain wall-pinning influencing the coercive force of the material and domain wall motion, which are responsible for the magnetizing behavior at low and medium applied external magnetic fields and the hysteresis losses [10]. The crystallographic texture of silicon steel influences its permeability and magnetic anisotropy. In the case of base-centered cubic (BCC) lattice, the easy axis of magnetization corresponds with <100> crystal orientation, i.e., when a crystal is magnetized along one of the <100> equivalent directions, the highest permeability is obtained as compared to any other magnetization directions {hkl}. Thus, the ideal crystallographic texture of NO electrical sheet is represented by a so-called Cube texture {100} <uv0> consisting of grains with the plane {001} parallel to the sheet surface and the <100> crystal directions uniformly distributed in the sheet plane [11,12,13,14]. Previous scientific works [15,16] have been focused on gradual improvement of microstructural and textural parameters of electro-technical steels during their hot rolling and final annealing by means of their chemical composition modification or introduction of innovative cold rolling and heat treatment processes.

It should be noted that NO electrical steel sheets are generally divided into two categories, namely the fully processed (i.e., fully finished) and semi-processed (i.e., semi-finished) grades. The semi-processed electrical steels are finished to their final thickness by the steel producer and then their final magnetic properties are achieved during the final heat treatment at the end-consumers after different cutting processes [17]. The fully finished NO steels are characterized by their final sheet thickness, microstructure, magneto-crystalline texture, and specific magnetic properties, which have been adjusted through hot band annealing, cold rolling, and final annealing of thin steel strip. The above-mentioned structural properties determine the steel’s final magnetic properties. The fully finished NO steels can be readily used for the final assembly by the equipment manufacturer [18]. In the most of electrical rotating devices, their core (i.e., rotor and stator) segments are commonly constructed from fully finished electrical steels. The segments of electrical motors often have complicated shapes, which are achieved by cutting of fully finished sheet. It is well-known that the preparation of laminated magnetic cores is associated with introduction of some residual (e.g., mechanical and/or thermal) stresses leading to the material final magnetic properties degradation. Thus, the performance of fully processed NO electrical steel laminations can, in particular, be seriously impaired by the cutting operations required to form the slotted stator core of rotating electrical machines [19,20,21]. Therefore, such segments are conventionally subjected to so-called stress relief heat treatment, which assures significant recovery and improvement of magnetic properties of NO silicon steel laminations. The changes in magnetic properties due to the annealing are closely related to thermally induced changes of residual stress, magnetic domain structure, microstructure, and crystallographic texture in the cutting edge zone [22].

Some previous scientific works [23,24,25] have confirmed a positive effect of conventional stress relief heat treatment on the magnetic properties improvement of punched fully finished silicon steels. Such an annealing process, which is normally used by end consumers in industrial conditions [24], includes the heating at very slow heating rate of 100 °C/1 h, followed by soaking for 1 h in the temperate range 700 °C–800 °C. The cooling of the materials is also realized at a very slow cooling rate. The whole conventional process (i.e., heating, annealing, and cooling) lasts more than 12 h. It is important to say that this conventional annealing process does not change the grain size of the samples because the grain growth kinetics is much too slow at 700 °C. During the process, only microstructural recovery takes place [23]. In other words, the initial steel microstructure and its texture do not change after such conventional heat treatment. It is because the fact that the material ate of fully finished steel does not possess any additional driving force, which would activate the grain growth under conventional annealing conditions. 

Some of our previous works [26,27,28,29] have clearly shown that substantial improvement of microstructural and textural parameters of electrical steels was possible by using the mechanism of strain-induced selective grain growth. The driving force of this mechanism is directly related to stored deformation energy of electrical steel subjected to temper rolling process. It is important to note that the obtained coarse-grained microstructure in our temper rolled samples was achieved as a result of secondary recrystallization annealing treatment using an extraordinarily high heating rate. Furthermore, the obtained experimental findings indicated that the annealing temperature has to be higher than 900 °C. After such dynamic heat treatments, the achieved microstructure and texture of the treated materials were found to have clearly positive effect on final magnetic properties, manifested by substantial decreases in core losses and coercivity. Even more, the proposed approach allowed not only improving the final magnetic parameters of NO steels, but it also enabled the reduction of the final production costs of end customers.

In the present work, the fully finished grade of NO silicon steel before the punching was subjected to bending rolling process in order to achieve a small plastic deformation. Subsequent heat treatment of the deformed strips was individually carried out in either conventional (slow heating rate) or unconventional (high heating rate) conditions. The main objectives of present study were focused on mutual comparison and discussion of the results obtained from microstructure, texture, and magnetic properties analyses of fully finished NO silicon steel sheets after initial introduction of fine plastic deformation and subsequent heat treatment in either conventional (long-term) or unconventional (dynamic) annealing conditions.

## 2. Materials and Methods 

The material investigated in the present work was a vacuum degassed fully finished NO steel (U.S. Steel Košice, Košice, Slovakia) of M530-50A grade with the following chemical composition in wt.%: Fe = 97.90, C = 0.004, Si = 1.32, Mn = 0.289, P = 0.039, Al = 0.346, other elements ~0.102%. A strip of 0.5 mm in thickness was taken from industrial line after final continuous annealing and marked as R1 samples. 

Then, these fully finished steel samples in the form of strips were variously thermo-mechanically treated under laboratory conditions. The description of individual material states of experimental samples is presented in Table 1.

**Figure 1:**
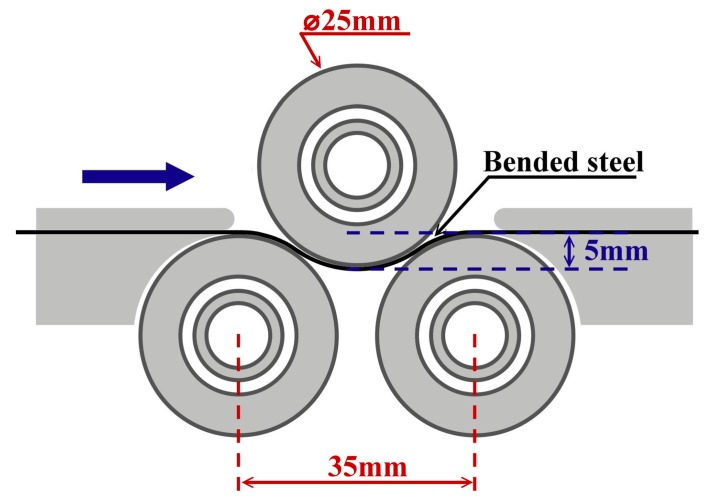
Three-roller bending system for mechanical treatment of experimental samples.

A soft plastic deformation has been introduced into experimental material by employing our in-house-designed three-roller system. A corresponding scheme of the bending deformation process is presented in Figure 1. Such a rolling system was used in order to achieve continuous bending of a plate under each straightening roll during the straightening. The diameter of each roller was 25 mm. The mechanical treatment used aimed at increasing the storage deformation energy of experimental samples without changing their thickness.

Both conventional (long-term) and unconventional (dynamic) heat treatments were individually applied to our experimental samples to study the grain growth phenomena in fully finished NO silicon steel. For individual analyses (i.e., microstructure, texture, and magnetic measurements analyses), experimental samples were heat treated in laboratory conditions using the electric resistance furnace Nabertherm RS 120/1000/13 (Nabertherm GmbH, Lilienthal, Germany). The long-term annealing treatment was carried out according to the EN 10 106 standard [30]. The samples were slowly heated up to 790 °C at a heating rate of about 100 °C/h. Subsequently, they were held at the temperature of 790 °C for 1 h and finally cooled at a rate of 200 °C/h. During the dynamic heat treatment, the samples were heated up to 950 °C at a heating rate of about 15–20 °C/s and then they were kept at this temperature for 5 min. Afterwards, the cooling process was realized at a rate of 5 °C/s. The annealing atmosphere for both the heat treatments was pure hydrogen with a dew point of 25 °C. The heating and cooling rates of the conventional long-term annealing process were controlled by internal thermocouples operated by digital controller of the furnace. In the case of dynamic heat treatment, the heating and cooling rates were calculated from the measured data of external thermocouple, which were joined to the annealed sample. In order to achieve such a high heating rate, the sample together with thermocouple was very quickly put into the furnace heated to 950 °C.

Microstructural analyses of selected representative samples corresponding to individual material states were performed using light optical microscope (LOM) OLYMPUS GX71 (OLYMPUS Europa Holding GmbH, Hamburg, Germany). In order to obtain statistic distribution of grain size, the selected microstructural states were processed by using free metallographic analysis software ImageJ (version J2). It enabled us to estimate the average grain size of the samples after individual heat treatments.

The selected representative samples were also used for texture analyses. The texture analyses were carried out by means of electron back-scattered diffraction (EBSD) method in the normal direction plane for each sample of 25 mm × 10 mm in size. The scanning electron microscope (SEM) JEOL JSM 7000F FEG (Jeol Ltd., Tokyo, Japan) with the EBSD detector Nordlys-I (HKL technology A/S, Hobro, Denmark) were used to perform the texture analysis. The obtained EBSD data were processed by the CHANNEL-5, HKL software package (Service pack 7).

The nano-hardness testing was carried out using Nano Indenter G200 (Agilent Technologies, Inc., Chandler, AZ, USA). These measurements were performed using Berkovich indenter on the surface of longitudinal cross-sections of experimental samples in both fully finished and bending rolled material states. The cross-section along the length of tested sample was divided into 9 imaginary lines. The nano-hardness measurements were performed along these individual lines mainly in the grains’ interiors at the distance minimally 5–10 µm from grains boundaries. The distance between individual indents was about 50 µm. The obtained values from around 10 indentations were averaged in order to evaluate the general hardness in each linear series parallel to the surface. 

The magnetic properties of experimental fully finished NO steels subjected to bending deformation and then heat treated according to both individual annealing schemes were investigated. These measurements were recorded on the toroid-shaped samples with outer diameter of 25mm and inner diameter of 15mm. The alternating current (AC) hysteresis loops were measured within the frequency range from 10Hz to 50Hz and at maximum induction of 1.5T by an AC/DC (direct current) Permeameter AMH-1K-S (Laboratorio Elettrofisico, Milan, Italy). The strip samples with planar dimensions of 80 mm × 30 mm (the longest dimension parallel to the rolling direction) were used for measurement of coercivity by Foerster Koerzimat 1.097 HCJ (Foerster, Reutlingen, Germany). Both types of the samples were prepared by electrical discharge machining using spark erosion machine EIR-EMO 2N (Emotek s.r.o., Nové Mesto nad Váhom, Slovak Republic). 

## 3. Results and Discussion

### 3.1. Nano-Indentation Measurements

During the bending of the thin steel sheet with a small radius, weak mechanical strains through the material thickness are generated and can be used for selective grain growth during the subsequent heat treatment. To investigate the accumulation of stored deformation energy and its dependence on the distance of the surface in small volumes in bending rolled samples, the nano-hardness tests were carried out.

**Figure 2:**
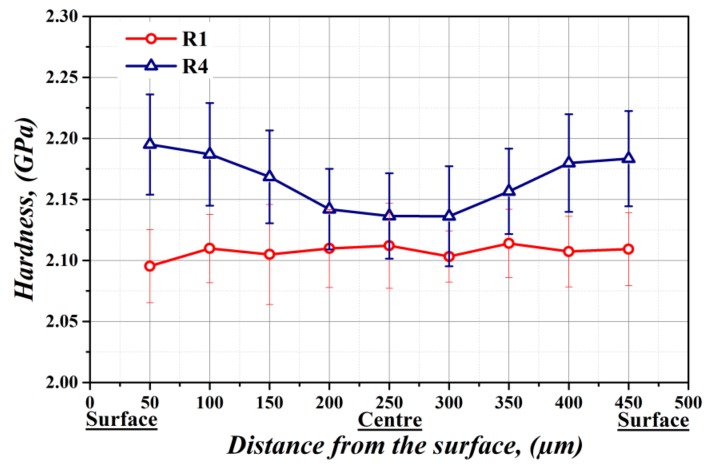
Cross-sectional nano-hardness dependence of experimental steel in its initial fully finished state (R1) and after subsequent bending deformation (R4).

It is well-known that steel sheets after plastic deformation possess accumulated deformation energy, which is stored in dislocation structures. It means that the steel subjected to mechanical treatment is characterized by increased dislocation density causing a certain material hardening, which can be detected via hardness measurements [31]. Based on this assumption, the nano-hardness measurements across the sample thickness were performed for R1 samples without any mechanical treatment (red line) and R4 samples processed by bending deformation (blue line) (see Figure 2). It is obvious from Figure 2 that the R1 sample without bending deformation exhibits uniform (i.e., almost unchanging) distribution of nano-hardness values through the whole steel sheet thickness. The average nano-hardness value was calculated to be 2.11 GPa. This value was considered the reference hardness of investigated fully finished steel defined by its chemical composition. In the case of the R4 sample after the bending rolling treatment, the corresponding nano-hardness dependence shows a significant hardness gradient from the sample surface to its central part (see blue line in Figure 2). The obtained dependencies clearly indicated significant increase in nano-hardness values in surface areas of plastically deformed R4 samples. The observed hardening in these areas reached more than 4%, compared to the central region. However, the central part of bending rolled samples was also characterized by some weak hardening, compared to the undeformed R1 samples. The obtained results can be related to inhomogeneous distribution of stress fields throughout the samples’ thickness during the bending deformation. Wang et al. [32] showed that thin plates subjected to bending deformation induced by roller systems with a small radius are characterized by plastic and elastic deformation regions throughout their cross-section. They have determined that the plastic deformation zones were localized near the plate surfaces and the elastic ones in its central part. It means that the mechanical stresses induced in the steel plate have bilinear distribution with maximal values in both subsurface regions and minimal value in the middle part. Qualitatively similar behavior was clearly confirmed by our nano-hardness measurements (Figure 2). It can be concluded, that the bending deformation leads to formation of certain gradient of stored deformation energy, without significant influence of initial thickness of the steel plate. The stored deformation energy is a necessary pre-condition for strain-induced grain boundary migration phenomena, which may take place after their thermal activation during subsequent secondary recrystallization heat treatment.

### 3.2. Microstructure Evolution 

**Figure 3:**
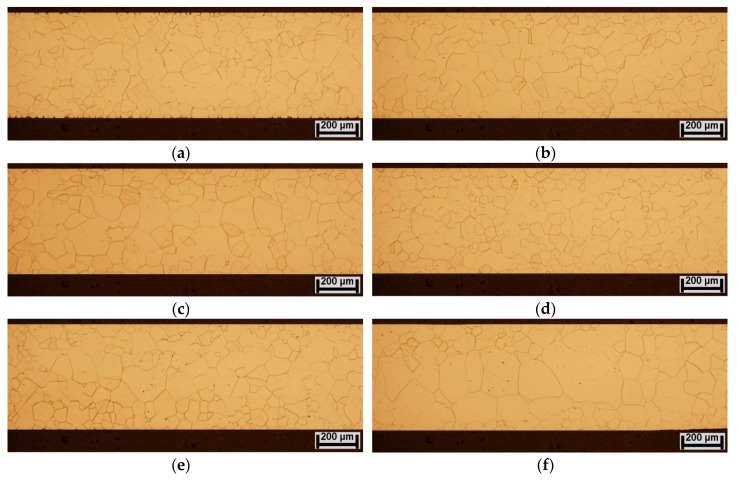
Microstructural variation of individual experimental samples of investigated non-oriented (NO) electrical steel corresponding to different material states: R1 (**a**); R2 (**b**); R3 (**c**); R4 (**d**); R5 (**e**); and R6 (**f**).

The microstructure evolution of experimental steel treated at different thermo-mechanical conditions is presented in Figure 3. The light-optical observations were performed on metallographic cross-sections of individually processed samples with their longitudinal part oriented parallel to the rolling direction. The observations show that all samples are characterized by monophasic ferrite microstructure with various distribution of grains. The initial microstructure of industrially fully finished NO steel (sample R1) is presented in Figure 3a. It can be seen that the sample is characterized by relatively fine-grained and homogeneous microstructure with average grain size of about 67.6 µm ± 3.5 µm. 

Microstructural characteristics of fully finished steel after conventional long-term heat treatment (sample R2) and after unconventional dynamic annealing (sample R3) are presented in Figure 3b,c, respectively. The microstructure of sample R2 after conventional annealing does not exhibit any specific microstructural changes. Its average grain size about 74 µm ± 3.8 µm. On the other hand, the sample R3, which was subjected to dynamic annealing treatment, demonstrates somewhat bimodal microstructure with average grain size of 80 µm ± 3.2 µm. The microstructure of experimental samples subjected to the bending deformation is presented in Figure 3d. Obviously, the microstructure of sample R4 is similar to the microstructure of sample R1. It can be concluded that our proposed mechanical treatment of fully finished steel does not affect its microstructural characteristics. In the case of bending rolled samples, R5 and R6, the grain growth behavior was also studied after their heat treatments in conventional (Figure 3e) and dynamic (Figure 3f) conditions, respectively. It can be clearly seen that the microstructures of these samples are quite different compared to the previous ones. In the case of sample R5 (Figure 3e), the microstructure exhibits notable improvement (i.e., increase) of average grain size in comparison with the initial microstructure (Figure 3a). Moreover, in the case of sample R6 (Figure 3f), the grain size increase is even more significant.

**Figure 4:**
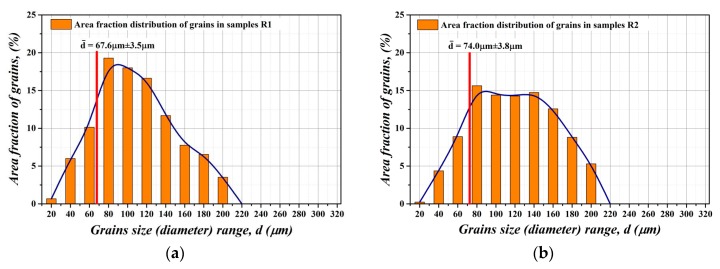

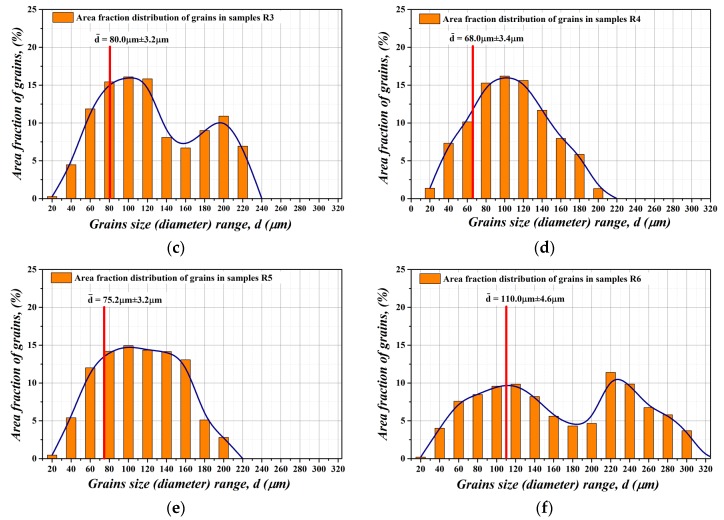
Grain size distribution characteristics of individual microstructures of investigated NO electrical steel corresponding to different material states: R1 (**a**); R2 (**b**); R3 (**c**); R4 (**d**); R5 (**e**); and R6 (**f**).

In order to quantify microstructural features of individual microstructures, their grain size distribution characteristics were evaluated (see Figure 4). From Figure 4 it can be seen that the R1 sample microstructure (Figure 4a) is characterized by the grain size variation in the range from 10 µm up to 200 µm. However, about 65% of R1 sample microstructure is formed of grains with their sizes in the range from 60 µm to 140 µm and about 18% of microstructure is occupied by the grains with their sizes up to 60 µm. The microstructure of the sample R2 is characterized by uniform distribution of ferrite grains with average grain size about 74 µm ± 3.8 µm. Also, 72% of the microstructure area is covered by grains with the size from 80 µm to 140 µm (see Figure 4b). The grain size distribution of R3 sample microstructure (Figure 3c) shows that 32% of microstructure is covered by grains in the range from 160 µm up to 220 µm. These large grains are mostly located in central part of the steel sheet cross-section. The sub-surface region is formed of grains that are similar to those occurring in R2 sample microstructure. Figure 4d shows the distribution of grain sizes of the bending rolled sample R4. It can be clearly seen that its grain size distribution is very similar to sample R1 (Figure 4a). Both these material states are characterized by same average grain size. It is possible to note that this kind of mechanical treatment does not influence the grain matrix parameters. The grain size distribution of R5 sample (Figure 4e) shows very similar characteristics as that for the R2 sample (Figure 4b). Thus, it can be concluded that conventional long-term heat treatment of bending rolled steel (R5 sample) practically does not affect its microstructure development. In the case of R6 sample processed by bending rolling and subsequent dynamic annealing treatment, the situation is quite different. The microstructure of R6 sample is characterized by bimodal grain size distribution with average grain size of 110 µm ± 4.6 µm (see Figure 3f and Figure 4f). Two microstructure regions can be clearly distinguished. The coarse-grained area with grain sizes ranging from 180 µm to 320 µm covers mostly the middle part of the sample and it occupies more than 50% of the sample cross-section. The fine-grained microstructure mostly occurs in the sub-surface regions of the sample. This part of microstructure is characterized by the grain size in the range from 60 µm to 140 µm.

**Figure 5:**
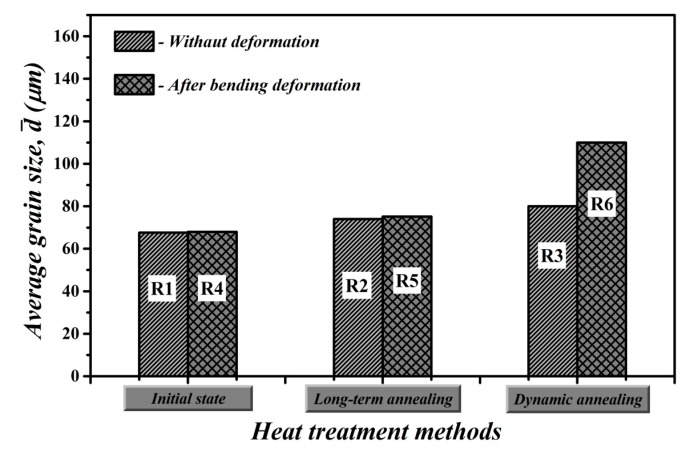
Average grain size of studied fully finished electrical steel in individual material states according to Table 1.

The comparison of average grain sizes of investigated samples in their individual material states is presented in Figure 5. It clearly demonstrates the effects of individual applied procedures (Table 1) on microstructure improvements of fully finished electrical steel. It can be concluded that our proposed unconventional treatment of the investigated steel, i.e., the bending rolling in combination with subsequent dynamic annealing at extraordinarily high heating rate, leads to significant increase in its average grain size (sample R6) in comparison with the initial industrially treated microstructure (sample R1). From Figure 5, it is apparent that the observed microstructural differences of individual experimental samples can be directly related to individual processing treatments, which were mutually different from each other. Conventional annealing of fully finished electrical steels is used in industrial conditions in order to remove residual stresses induced in material during cutting operations. The stress relief heat treatment is realized at slow heating rates and maximum annealing temperature of about 800 °C. The scheme of long-term heat treatment is very similar to the process of secondary recrystallization heat treatment, which is commonly used for the semi-finished electrical steels [17]. However, in contrast to semi-finished steel, which is characterized by pronounced microstructural evolution, the microstructure of fully finished steel does not exhibit any significant changes after the heat treatment (see Figure 3b). This observation can be easily explained by the fact that fully finished steel does not possess the necessary storage deformation energy, which represents the main driving force for strain-induced grain boundary migration (SIBM) [33]. In our previous works [26,27], it has been shown that pronounced selective grain growth could be achieved in temper-rolled NO steel subjected to small plastic deformation (about 2%–6%) and subsequent dynamic heat treatment using a very high heating rate. The light optical images in Figure 3a–c have clearly shown that in the case of fully finished steels that were subjected to the heat treatment in either conventional long-term (sample R2) or unconventional short-term dynamic annealing conditions (sample R3), the occurrence of grain growth was rather limited. This behavior fairly agrees with the well-known hypothesis that some minimum level of stored deformation energy is needed for activation of grain growth. The effect of dynamic annealing conditions on the columnar grain growth was investigated by Sidor and co-workers [34]. They showed that rapid heating is responsible for the occurrence of temperature gradient along the normal of the sheet thickness and this gradient might represent additional driving force for the grain growth. Other research works [35,36] have clearly indicated a beneficial effect of rapid annealing on the increase in average grain size and significant improvement of crystallographic texture of electrical steel after strip-casting and cold rolling process.

In the case of fully finished steel that was subjected to plastic deformation via bending rolling process, the microstructural evolution during subsequent heat treatment was varied independent of the used annealing conditions. In the case of conventional (long-term) annealing process of bending rolled fully finished steel (sample R5, Figure 3e), the microstructure did not exhibit any significant changes compared to conventionally annealed microstructure without prior bending rolling (sample R2, Figure 3b). A significant microstructural evolution occurred only in the bending rolled microstructure subjected to dynamic annealing treatment (sample R6, Figure 3f). The observed grain growth has been obtained by means of combination of two driving forces. One of them is related to the gradient of stored deformation energy, which was confirmed by nano-indentation measurements (see Figure 2) and the second one to the strong gradient of heat flow, which has been achieved thanks to the high heating rate during the dynamic heat treatment.

### 3.3. Texture

**Figure 6:**
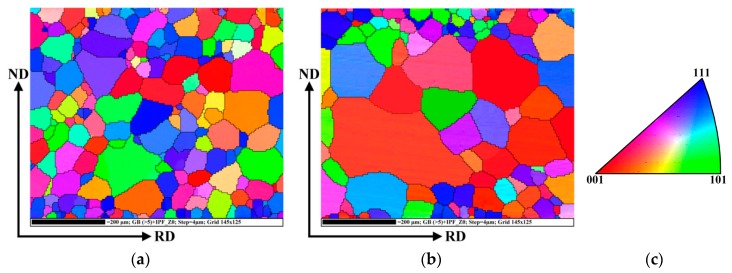
IPF representation of grain crystallographic orientations of investigated NO electrical steel in initial fully finished state—sample R1 (**a**) and after bending rolling and subsequent dynamic heat treatment—sample R6 (**b**). The key for the identification of crystallographic orientation is located on the right (**c**).

In order to investigate the texture evolution of selected samples with most significant improvement of microstructure, detailed EBSD analyses were performed. The crystallographic orientations of grain structures of the investigated samples in original fully finished state and those subjected to both bending deformation and dynamic heat treatment are presented by Inverse Pole Figure (IPF) maps in Figure 6a,b, respectively.

The coloured maps in Figure 6 characterize crystallographic orientations of all individual grains located in cross-sectional plane of the steel sheet. It can be clearly seen that the individual samples differ from each other not only by their microstructural parameters (i.e., grain size) but also by their crystallographic texture. In the case of sample R1, the dominant crystallographic orientation is so-called deformation texture which is characterised by the least effective magnetization direction <111> parallel to the normal of the sheet plane (the blue-coloured grains). The presence of this texture is rather undesirable in soft magnetic materials because it deteriorates their final magnetic properties. On the other hand, the sample R6 is characterized by pronounced cube crystallographic texture (the red-coloured grains) which is characterized by the easy magnetization direction <100>. In addition, Figure 6b clearly shows that the rotated cube crystallographic orientation is mostly related to the grains which were formed after the dynamic heat treatment and are localised in the central part of the cross-section. These results of selective grain growth correspond well with the results presented in Figure 3e. 

**Figure 7:**
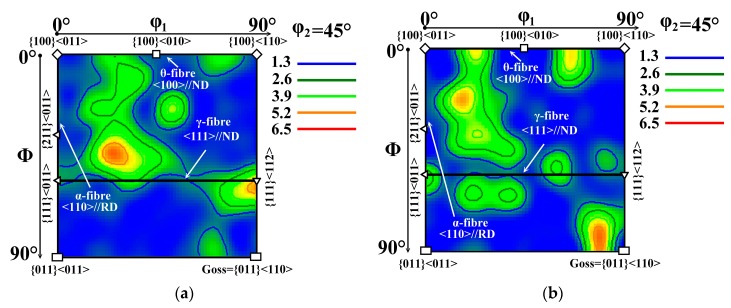
ODF sections taken at φ_2_ = 45° representing the through-thickness textures evolved in investigated samples R1 (**a**) and R6 (**b**).

The crystallographic texture of investigated samples can be interpreted by orientation fibres in the Euler space. The most relevant fibres for the NO electrical steels are θ-fibre (<100>//ND), γ-fibre (<111>//ND), and α-fibre (<110>//RD) [10] which are presented by means of orientation distribution function (ODF). The ODF sections taken at φ_2_ = 45° for the investigated samples R1 and R6 are shown in Figure 7a,b, respectively. 

**Figure 8:**
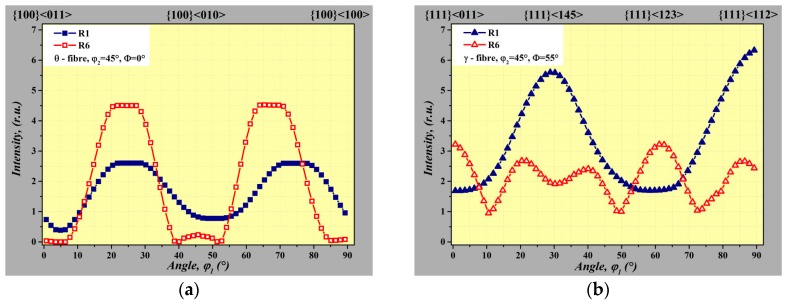
The variation of orientation density along θ-fibre (**a**) and γ-fibre (**b**) in NO electrical steel in fully finished material state—sample R1 (blue curve) and after bending deformation followed by dynamic annealing—sample R6 (red curve).

The development of the θ-fibre and γ-fibre of the investigated samples is presented in Figure 8. The texture of the sample R1 has the maximum intensity at the {111}<145> and {111}<112> component which corresponds to the γ-fibre (see Figure 8b). The θ-fibre exhibits the lower intensity of rotated cube with enhanced {100}<110> orientation at φ_1_ = 25° and 75°, see Figure 8a (blue line). The pronounced evolution of rotation cube was detected in sample R6. Here, θ-fibre is expressed by dominant intensity of cube crystallographic orientation with maximum intensity at φ_1_ = 20° and 65°. The intensity of deformation texture is presented by γ-fibre in Figure 8b (red curve). This figure clearly shows significant suppression of deformation texture component {111}<145> and {111}<112> of the sample R6 subjected to the bending deformation and dynamic annealing.

The evaluation of texture components of investigated fully finished steel clearly shows that the plastic deformation induced by mechanical bending in combination with dynamic annealing have a very positive effect on the development of desired microstructure exhibiting coarse grains with rotated cube crystallographic orientation. A small deformation gradient through the sheet cross-section achieved by bending rolling and detected by our nano-indentation measurements (Figure 2), provides the necessary stored deformation energy, serving as primary driving force for the selective grain migration [33].

As a result, the sample R6 after bending rolling and subsequent dynamic heat treatment shows significant selective grain growth of some specific grains of the initial grain matrix (Figure 6, Figure 7 and Figure 8). However, some other works [37,38] reported that the stored deformation energy may vary different texture components of polycrystalline materials that were subjected to mechanical deformation. It has been found experimentally that the plastic deformation which is stored in dislocation structures is distributed heterogeneously between the grains and depends on their crystallographic orientation, at least on orientations families defined by the crystallographic plane lying parallel to the sheet plane. In vacuum degassed ferrite steels, the magnitude of stored deformation energy of individual grains orientations can be characterized as follows: E_{111}_ > E_{112}_ > E_{100}_, where the lower index describes the crystallographic orientation of grains. It can be indicated that after applied mechanical treatment, the grain structures with {111}<112> crystallographic component may accumulate more deformation energy than rotated cube grains. It can be reasonably supposed that between the neighboring grains with various crystallographic orientations, a creation of some small gradient of stored deformation energy is related to the lattice defects such as dislocations, which were induced by the applied mechanical stress. Moreover, according to the thermodynamics principle, the gradient of stored energy representing a driving force for SIBM should enable promoting the migration of grains with less internal energy [39]. Taking into account all the mentioned results and considerations, it can be concluded that the fully finished electrical steels subjected to bending deformation are characterized by inhomogeneous distribution of stored deformation energy between the grains, which basically depends on their crystallographic orientation and arrangement through the sheet thickness. The stress disparity on some grain boundaries in combination with a high temperature gradient during the stress relief annealing leads to the achievement of selective growth of grains with appropriate rotated cube crystallographic orientation. This fact is directly confirmed by the results obtained in the present investigation showing adequate correlation between microstructure (Figure 3) and texture (Figure 6, Figure 7 and Figure 8) characteristics. The comparison of the microstructural and textural states of different samples subjected to stress relief at conventional (long-term) and unconventional (dynamic) annealing conditions clearly indicate that the temperature gradient creates appropriate conditions in uniformly bending rolled polycrystalline silicon steels for the evolution of microstructure with preferential rotated cube crystallographic orientation.

### 3.4. Magnetic Measurements

**Figure 9:**
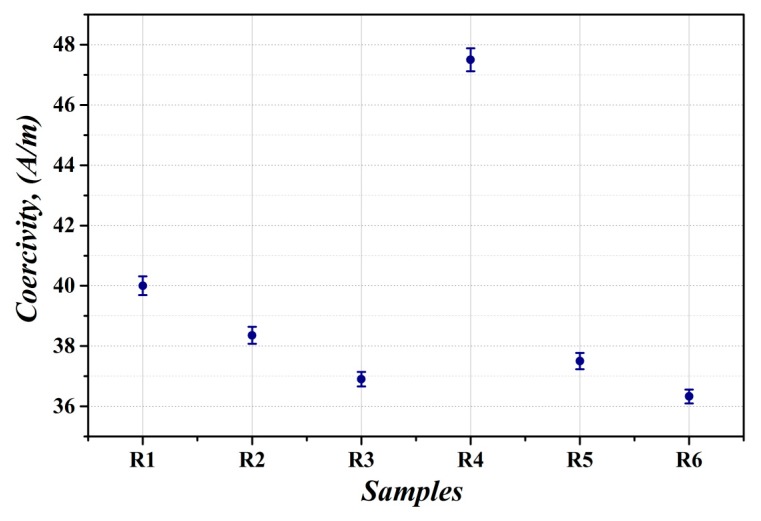
The measured coercivity of fully finished electrical steel in individual material states according to Table 1.

The evolution of microstructural, sub-structural, and textural characteristics of the investigated steel material states has its direct influence on their final magnetic properties, namely coercivity and power losses. The magnetic measurements were carried out for all experimental samples in direct current (DC) and alternating current (AC) magnetic field. The dependence of coercivity of experimental samples in individual material states is presented in Figure 9. It clearly indicates that the investigated samples differ from each other not only by microstructural, textural, and mechanical stress characteristics, but also by their coercivity. The initial value of coercivity corresponding to fully finished sample R1 is about 40.0 A/m ± 0.31 A/m. The samples R2, R3, R5, and R6 show clearly lower coercivity values than the sample R1. Very low coercivity values were exhibited by the samples R3 and R6, which indicated a crucial influence of dynamic heat treatment on soft magnetic characteristics improvement.

The minimum value of coercivity of about 36.33 A/m ± 0.23 A/m was obtained for the sample R6, which was subjected to the bending deformation with subsequent dynamic annealing at extraordinarily high heating rate. The obtained results indicate that the heat treatment of fully finished steels at dynamic heating conditions has a much more pronounced effect on their coercivity than conventional stress relief annealing carried out using relatively slow heating rate. The highest value of coercivity (47.5 A/m ± 0.38 A/m) was obtained for the sample R4. This result corresponds well with performed nano-indentation measurements and it can be related to notable increase in mechanical stresses in the studied steel after the bending deformation. 

**Figure 10:**
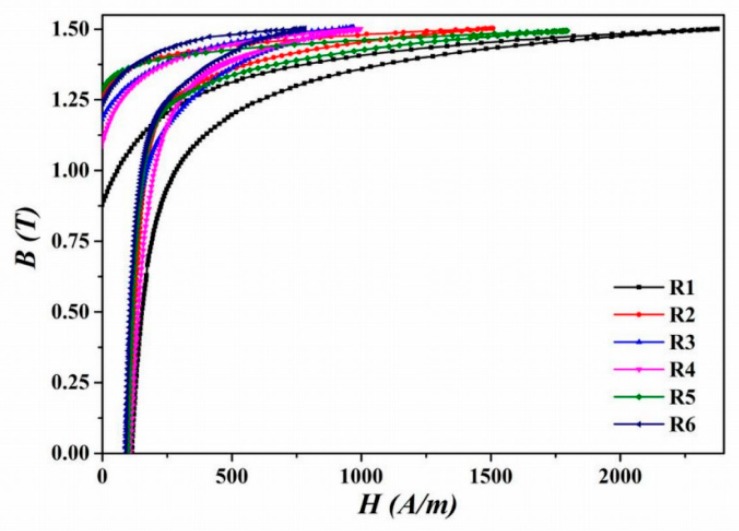
The B-H hysteresis loops recorded for the studied samples at 50Hz in the first quadrant.

The evolution of power losses of individual experimental samples, prepared in RD, was measured in AC magnetic field at a frequency of 50 Hz. Figure 10 shows an overview of the first quadrant of recorded hysteresis loops of the studied samples. 

The positive influence of annealing and bending processes on the position of the hysteresis loops knee is evident compared with hysteresis loop of sample R1. The significant decrease in the slope of the ascending part of the hysteresis loop for sample R1 is at 0.6 T and for other samples at this same point over 1.0 T. It can be explained by easier domain wall displacement in annealed or bended materials due to the generation of suitable grain texture. The external applied field H_max_ for B_max_ = 1.5 T is decreasing with dynamic annealing conditions. The sample R6 acquires B_max_ =1.5 T at the lowest magnetic field, 770 A/m. The R6 sample material state obtained by combination of bending rolling and dynamic heat treatment is also characterized by the steepest hysteresis loop, which is related to the increase in microstructure average grain size and the high intensity of θ-fiber texture components. 

**Figure 11:**
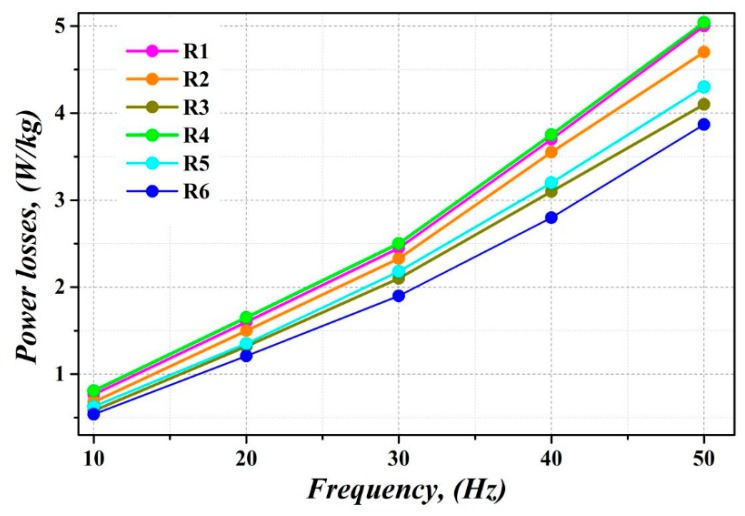
Power losses as a function of magnetizing frequency in studied samples at B_max_ = 1.5 T.

It is important to note that the magnetic properties of electrical steels are very sensitive to the value of the frequency of external AC magnetic field. It is related to the magnetic loss components, which are called excess loss. These losses are strongly depended on the movement of magnetic domains structures during the change of magnetic field at different frequency. Therefore, all experimental samples were subjected to the magnetic measurements in the AC magnetic field with the range of frequency from 10 Hz up to 50 Hz. Corresponding curves describing the dependence of power losses of the experimental samples on the magnetic field frequency range are presented in Figure 11. Here, overall better response of the samples heat treated after the bending deformation at dynamic annealing conditions (Sample R6—blue line) is put in evidence. The power losses are decreased thanks to coarse grains (see Figure 3e), which are mostly characterized by appropriate rotated cube crystallographic orientation (see Figure 6b). The highest value of losses was measured for the sample R4, which can be explained by the presence of mechanical stresses, which were induced into the steel by the applied bending rolling. On the other hand, the dynamic heat treatment of experimental samples R3 and R6 (i.e., the samples without or with bending deformation), significantly reduces their power losses (see Figure 11). This observation can be related to the increase in average grain size of these samples after the dynamic heat treatment. The reduction of power losses of the samples R2 and R5 subjected to conventional stress relief annealing using slow heating rate is not as expressive as in samples R3 and R6. 

It can be clearly seen that the measurements of power losses at varying magnetizing frequency (Figure 11) perfectly correspond with the results of coercivity measurements in the DC magnetic field (Figure 9). Moreover, the curves presenting the dependence of power losses on different frequencies of AC magnetic field show that the bending rolled sample, heat treated in dynamic conditions, shows more than 17% decrease in power losses at 50 Hz in comparison with the samples treated by conventional heat treatment leading only to stress relief without additional grain growth. The divergence of the power losses lines occurs at higher frequencies due to excess loss as a part of the dynamic losses responding for eddy current loss caused by domain wall displacement.

## 4. Summary and Conclusions 

In present work, the effects of different heat treatment conditions and bending rolling deformation were studied with respect to microstructural evolution, crystallographic texture, and magnetic loss characteristics of fully finished non-oriented electrical steel. In order to achieve the effect of strain-induced selective grain boundary motion, the investigated steel was subjected to bending rolling prior to heat treatment. The annealing of plastically deformed samples was individually carried out by either conventional long-term or unconventional dynamic stress relief heat treatment. It has been clearly demonstrated that the used bending deformation in combination with subsequent dynamic heating process results in substantial microstructure improvement characterized by coarsened grains with enhanced intensity rotated cube texture. The main observations and conclusions can be summarized as follows:

- The nano-indentation measurements have clearly shown that the bending rolled strips are characterized by bilinear distribution of nano-hardness through the sheet thickness. The maximal nano-hardness values were measured in both sub-surface regions, whereas the minimal value in the sheet cross-section middle part. This observation is related to the high intensity of plastic deformation in both the sub-surface regions and only elastic stress in the middle part of the samples after the bending rolling. Thus, it can be concluded that the bending deformation enabled achieving the gradient of stored deformation energy, without changing the initial thickness of the steel plate.
- The heat treatment at dynamic annealing conditions of fully finished NO silicon steels subjected to the mechanical bending deformation leads to apparent increase in average grain size of the obtained microstructure. The evolution of coarse-grained microstructure is related to the strain-induced grain boundary migration mechanism under the influence of significant temperature gradient through the steel sheet cross-section.
- The texture measurements have clearly shown that the unconventional dynamic annealing of bending rolled experimental samples had a clearly positive impact on the evolution of their crystallographic orientation. It has been shown that coarse-grained matrix obtained after the heat treatment with high heating rate was characterized mostly by the grains with desirable rotated cube crystallographic orientations.
- The magnetic measurements of fully finished samples in DC and AC magnetic field conditions have clearly indicated that the evolved microstructures and textures of the strips, obtained by application of bending deformation and heat treatment using two different procedures, are directly responsible for their final magnetic characteristics. The power losses data have clearly shown that the investigated steel treated according to our innovative approach exhibited more than 17% decrease in watt losses in comparison with the material treated by conventional stress relief heat treatment without activation of grain growth.

## Figures and Tables

**Table 1:** Description of individual material states of experimental samples.

Samples	Treatment Conditions
R1	The steel taken from the industrial line in fully finished state.
R2	The R1 samples treated according to conventional stress-relief annealing.
R3	The R1 samples heat treated at dynamic conditions with high heating rate
R4	The R1 samples which were subjected to the bending deformation.
R5	The R4 samples treated according to the conventional stress-relief annealing.
R6	The R4 samples heat treated at dynamic conditions with high heating rate.
